# Medical end-of-life practices in Swiss cultural regions: a death certificate study

**DOI:** 10.1186/s12916-018-1043-5

**Published:** 2018-04-20

**Authors:** Samia A. Hurst, Ueli Zellweger, Georg Bosshard, Matthias Bopp, for the Swiss Medical End-of-Life Decisions Study Group, Karin Faisst, Felix Gutzwiller, Christoph Junker, Milo Puhan, Margareta Schmid

**Affiliations:** 1 Institute for Ethics, History, and the Humanities, Geneva University Medical School, 1211 Genève, Switzerland; 2 Epidemiology, Biostatistics and Prevention Institute, University of Zurich, Hirschengraben 84, CH-8001 Zürich, Switzerland; 3 Clinic for Geriatric Medicine, Zurich University Hospital, and Center on Aging and Mobility, University of Zurich and City Hospital Waid, Rämistrasse 100, 8091 Zürich, Switzerland

## Abstract

**Background:**

End-of-life decisions remain controversial. Switzerland, with three main languages shared with surrounding countries and legal suicide assistance, allows exploration of the effects of cultural differences on end-of-life practices within the same legal framework.

**Methods:**

We conducted a death certificate study on a nationwide continuous random sample of Swiss residents. Using an internationally standardized tool, we sent 4998, 2965, and 1000 anonymous questionnaires to certifying physicians in the German-, French-, and Italian-speaking regions.

**Results:**

The response rates were 63.5%, 51.9%, and 61.7% in the German-, French-, and Italian-speaking regions, respectively. Non-sudden, expected deaths were preceded by medical end-of-life decisions (MELDs) more frequently in the German- than in the French- or Italian-speaking region (82.3% vs. 75.0% and 74.0%, respectively), mainly due to forgoing life-prolonging treatment (70.0%, 59.8%, 57.4%). Prevalence of assisted suicide was similar in the German- and French-speaking regions (1.6%, 1.2%), with no cases reported in the Italian-speaking region. Patient involvement was smaller in the Italian- than in the French- and German-speaking regions (16.0%, 31.2%, 35.6%). Continuous deep sedation was more frequent in the Italian- than in the French- and German-speaking regions (34.4%, 26.9%, 24.5%), and was combined with MELDs in most cases.

**Conclusion:**

We found differences in MELD prevalence similar to those found between European countries. On an international level, MELDs are comparably frequent in all regions of Switzerland, in line with the greater role given to patient autonomy. Our findings show how cultural contexts and legislation can interact in shaping the prevalence of MELDs.

## Background

In many countries worldwide, there is persistent controversy surrounding end-of-life decisions, particularly in relation to assisted suicide and euthanasia. Debates regarding the legal status of such decisions assume that legislative differences [1] and care settings [2, 3] largely determine international variation in prevalence. Countries where these decisions are legal and where several cultures co-exist, such as Switzerland, Belgium, the US, and more recently Canada, thus present an important opportunity to explore the role of legal and cultural frameworks for variations of end-of-life decisions.

Switzerland allows suicide assistance if it is offered without selfish motive and even when it is practiced by non-physicians [4], without recognizing an entitlement to such assistance [5]. In contrast to the Netherlands, Belgium, and Luxemburg, but similarly to the US states allowing suicide assistance, euthanasia is not legal [6–8]. “Suicide tourism” toward Switzerland has influenced end-of-life debates in countries such as Germany, the UK, the US, and Canada, from which many assisted suicide candidates originate [9].

Switzerland has four official languages, German, French, Italian, and Romansh, within defined geographical areas. The language regions share many cultural features with the respective neighboring countries, offering an opportunity to explore the effects of cultural differences on end-of-life practices within the same legal context [10]. Reliable population-level data on this field has only been collected once in Switzerland in 2001, within the EURELD study [11]. However, the Swiss sample was limited to the German-speaking region. Other studies suggest that French physicians’ support for legalizing euthanasia could be greater [12], and German physicians’ lesser [13], than their Swiss counterparts. The ETHICUS study showed an increase in frequency in withdrawal of life-sustaining treatment from South to North Europe [14]. In the EURELD study, however, Sweden and Denmark showed lower prevalence than Belgium and the Netherlands, and much lower prevalence than (German-speaking) Switzerland [15]. Further, data from Belgium suggests that cultural differences between regions affect euthanasia practices within a country [2]. In Switzerland, substantial variations were described in physicians’ attitudes between language regions, the most striking being a reluctance of Italian-speaking doctors against any kind of end-of-life decisions – in close similarity to Italy [16].

In 2013, we performed a follow-up study of the Swiss part of the EURELD study using the same methodology and largely the same questionnaire [11], but including the French- and Italian-speaking regions. Comparative data related to the German-speaking region of Switzerland in 2001 and 2013 have been published recently [17, 18]. This paper presents cross-sectional data of the different language regions of Switzerland on medical end-of-life decisions (MELDs).

## Methods

### Participants

Study methods have been described in more detail elsewhere [17, 18]. A continuous random sample of death certificates of residents aged 1 year or older was selected on a weekly basis by the Swiss Federal Statistical Office. We differentiated between German- (71.6% of total population), French- (23.6%), and Italian-speaking (4.4%) areas. Since Romansh, the fourth national language, is only spoken by less than 1% of the national population and its geographical area is not contiguous, it was included into the German-speaking region. Taking into account the smaller population size, the French- and Italian-speaking regions were oversampled. The sample size was chosen in order to obtain reliable data from all three language regions while limiting the risk that some physicians in the smaller language regions received too many questionnaires. In total, 21.3%, 41.1%, and 62.9% of registered deaths were respectively sampled among residents of the German-, French-, and Italian-speaking regions of Switzerland, and certifying physicians were invited to participate. Between August 1, 2013, and January 31, 2014, we sent 4998, 2965, and 1000 questionnaires in weekly batches to the three respective language regions. The last completed questionnaire arrived on June 11, 2014.

### Survey tool

If death was not sudden and unexpected, the case was considered eligible for questions regarding end-of-life decisions. In such cases, physicians were asked whether they had (1) withheld or withdrawn a probably life-prolonging medical treatment taking into account or explicitly intending to hasten the patient’s death; (2) intensified the alleviation of pain and/or symptoms with drugs taking into account or partly intending to hasten the patient’s death; or (3) prescribed or administered a drug with the explicit intention of ending the patient’s life (physician-assisted death). For this study, we categorized cases as a physician-assisted death when a positive response was given to question (3), irrespective of answers to questions (1) and (2). Positive answers to question (3) were categorized as follows:

(3a) “assisted suicide”, if patients self-administered the drug to end their life;

(3b) “euthanasia”, if somebody else administered the drug and the question regarding explicit request of the patient was answered affirmatively;

(3c) “ending of life without the patient’s explicit request”, if the question regarding explicit request of the patient was not answered affirmatively.

To evaluate continuous deep sedation, physicians were asked if the patient received medicines to maintain them in a continuous deep sedation or coma until death.

The survey tool was translated into French and Italian, back translated for quality control, and checked by bilingual individuals. The final questionnaire (four pages) is available upon request.

### Human participant protection

To guarantee anonymity, physicians were requested to return questionnaires to the Swiss Academy of Medical Sciences. Questionnaires were only forwarded to the investigators at the Epidemiology, Biostatistics, and Prevention Institute of the University of Zurich (then Institute of Social and Preventive Medicine) after confirmation that the code key had been deleted for this case. Questionnaire return was considered to imply consent to participate. The study was declared exempt from ethics review by the Zurich Cantonal Ethics Board (KEK-StV-Nr. 23/13).

### Data analysis

Questionnaires were scanned and all data were weighted to adjust for region-, age-, and sex-specific differences in response rates. Weighted percentages and 95% confidence intervals for the comparison of the three language regions were calculated using STATA 13.1 survey tables for weighted data (StataCorp LP, College Station, TX, US).

## Results

### Sample

Of 8963 mailed questionnaires, 3173 (63.5%), 1538 (51.9%), and 617 (61.7%) were returned from the German-, French-, and Italian-speaking regions of Switzerland, respectively, which is comparable to other research using this method [6].

### Medical end-of-life decisions (MELDs)

Non-sudden, expected deaths were preceded by at least one MELD in a majority of cases, more frequently (82.3%) in the German- than in the French- (75%) or Italian-speaking (74%) regions (Table 1). Focusing on the most explicit practice, forgoing life-prolonging treatment was the most frequent MELD in the German-speaking region and intensified alleviation of symptoms the most frequent in the French-speaking region (49.3% of non-sudden expected deaths and 39.8%, respectively), with both being similarly frequent in the Italian-speaking region (34.8% and 37.4%, respectively). Assisted suicide was reported in 1.6% and 1.2% of non-sudden expected deaths in the German- and French-speaking regions, with no cases reported in the Italian-speaking region in our sample.

**Table 1:** Prevalence of medical end-of-life practices^a^ in Switzerland 2013, by language region

Regions	German-speaking		French-speaking		Italian-speaking	
Number of non-sudden expected deaths (eligible for end-of-life decision)	*N* = 2256		*N* = 992		*N* = 430	
	%^b^	95% CI	%^b^	95% CI	%^b^	95% CI
No end-of-life practice	17.7%	(16.2–19.3)	25.0%	(22.4–27.9)	26.0%	(22.0–30.3)
Forgoing life-prolonging treatment	49.4%	(47.3–51.4)	31.6%	(28.8–34.6)	34.8%	(30.4–39.5)
- taking into account hastening of death^c^	6.4%	(5.4–7.5)	5.2%	(4.0–6.7)	4.7%	(3.0–7.1)
- intending hastening of death^d^	43.0%	(40.9–45.0)	26.5%	(23.8–29.3)	30.1%	(26–34.7)
Intensified alleviation of pain/symptoms	29.8%	(28.0–31.7)	39.8%	(36.8–42.9)	37.4%	(33.0–42.1)
- taking into account hastening of death^e^	26.9%	(25.1–28.8)	36.6%	(33.7–39.7)	33.8%	(29.5–38.4)
- partly intending hastening of death^f^	2.9%	(2.3–3.7)	3.2%	(2.3–4.5)	3.6%	(2.2–5.8)
Physician-assisted death	3.1%	(2.5–3.9)	3.5%	(2.5–4.8)	1.8%	(0.9–3.6)
- Assisted suicide^g^	1.6%	(1.1–2.2)	1.2%	(0.6–2.1)	–	
- Euthanasia^h^	0.5%	(0.3–0.9)	0.5%	(0.2–1.2)	0.5%	(0.1–1.8)
- Ending of life without the patient’s explicit request^i^	1.1%	(0.8–1.6)	1.9%	(1.2–2.9)	1.4%	(0.6–3.0)

^a^If several practices were combined, the most explicit action was decisive; e.g., combinations of physician-assisted death with forgoing life-prolonging treatments or intensified alleviation of pain and symptoms were categorized under physician-assisted death

^b^100% = all non-sudden expected deaths; percentages weighted to region-sex-age-specific response rates

^c^Affirmative answer to the question, “Did you or another physician withhold or withdraw a medical treatment while taking into account the possible hastening of death?”

^d^Affirmative answer to the question, “Did you or another physician withhold or withdraw a medical treatment with the intention to hasten death?”

^e^Affirmative answer to the question, “Did you or another physician intensify the alleviation of pain and/or symptoms while taking into account the possible hastening of death?”

^f^Affirmative answer to the question, “Did you or another physician intensify the alleviation of pain and/or symptoms partly with the intention to hasten death?”

^g^Affirmative answer to the question, “Was death the consequence of the use of a drug that was prescribed or supplied by you or another physician with the explicit intention of enabling the patient to end his or her life?”

^h^Affirmative answer to the question, “Was death the consequence of the use of a drug that was prescribed or supplied by you or another physician with the explicit intention of hastening the patient’s death?” AND affirmative answer to the question: “Was this decision made at the explicit request of the patient?”

^i^Affirmative answer to the question, “Was death the consequence of the use of a drug that was prescribed or supplied by you or another physician with the explicit intention of hastening the patient’s death?” AND no affirmative answer to the question: “Was this decision made at the explicit request of the patient?”

MELDs were combined in approximately half of the cases (Table 2). When all cases including a decision to forgo life-prolonging treatment were considered, the intention to shorten life was more frequent than only taking this into account in the German-speaking region (44.2% vs. 25.8%), while there were no statistically significant differences in the other two regions. Intensified alleviation of symptoms was similarly prevalent in all language regions. There was no intention to shorten life in most cases of alleviation of pain and symptoms; however, in a minority of cases, shortening of life was partly intended (more often in the German- and Italian- than in the French-speaking region).

**Table 2:** Forgoing life-prolonging treatment and intensified alleviation of pain and symptoms, Switzerland 2013, by language region

Regions	German-speaking		French-speaking		Italian-speaking	
Number of non-sudden expected deaths (eligible for end-of-life decision)	*N* = 2256		*N* = 992		*N* = 430	
	%^a^	95% CI	%^a^	95% CI	%^a^	95% CI
Forgoing life-prolonging treatment						
Total	70.0%	(68.1–71.9)	59.8%	(56.7–62.8)	57.4%	(52.7–62.0)
- taking into account hastening of death	25.8%	(24.0–27.6)	32.1%	(29.3–35.1)	25.7%	(21.8–30.1)
- intending hastening of death	44.2%	(42.2–46.3)	27.7%	(25.0–30.6)	31.7%	(27.5–36.3)
- not combined with other medical end-of-life practice (1)	17.3%	(15.8–18.9)	12.5%	(10.6–14.7)	10.2%	(7.7–13.5)
- combined with intensified alleviation of pain/symptoms only	51.2%	(49.1–53.2)	45.0%	(41.9–48.1)	45.4%	(40.7–50.1)
- ditto, only intended forgoing treatment (2)	32.0%	(30.1–34.0)	19.2%	(16.8–21.7)	24.6%	(20.7–28.9)
- combined with physician-assisted death	1.5%	(0.1–2.1)	2.3%	(1.5–3.5)	1.8%	(0.9–3.6)
Intensified alleviation of pain/symptoms						
Total	63.4%	(61.4–65.3)	61.4%	(58.3–64.4)	63.8%	(59.1–68.2)
- taking into account hastening of death	51.7%	(49.7–53.8)	53.8%	(50.7–56.9)	48.8%	(44.1–53.5)
- partly intending hastening of death	11.6%	(10.4–13.0)	7.6%	(6.1–9.4)	15.0%	(11.9–18.8)
- not combined with other medical end-of-life practice (3)	10.7%	(9.5–12.0)	14.0%	(12.0–16.4)	16.6%	(13.4–20.5)
- combined with forgoing life-prolonging treatment only	51.2%	(49.1–53.2)	45.0%	(41.9–48.1)	45.4%	(40.7–50.1)
- ditto, only non-intended forgoing treatment (4)	19.1%	(17.6–20.8)	25.8%	(23.2–28.7)	20.8%	(17.2–24.9)
- combined with physician-assisted death	1.5%	(0.1–2.1)	24.0%	(1.6–3.6)	1.8%	(0.9–3.6)

^a^100% = all non-sudden expected deaths; percentages weighted to region-sex-age-specific response rates

Data in this table include cases in which more than one end-of-life decision were taken

Forgoing life-prolonging treatment as most explicit end-of-life decision (cf. Table 1): (1) + (2)

Intensified alleviation of pain/symptoms as most explicit end-of-life decision (cf. Table 1): (3) + (4)

### Continuous deep sedation (CDS)

Death was preceded by medication to bring about CDS in many non-sudden, expected deaths (Table 3). This was more frequent in the Italian-speaking region than in the German- and French-speaking regions (34.4% vs. 24.4% and 26.9%). CDS were combined with MELDs in most cases. Deaths preceded by CDS without a MELD were slightly, but statistically significantly more frequent in the Italian- than in the German-speaking region (with intermediate prevalence in the French-speaking region).

**Table 3:** Continuous deep sedation in Switzerland 2013, by language region

Regions	German-speaking		French-speaking		Italian-speaking	
Non-sudden expected deaths	*N* = 2256		*N* = 992		*N* = 430	
	%^a^	95% CI	%^a^	95% CI	%^a^	95% CI
Continuous deep sedation until death (CDS)	24.5%	(22.3–26.3)	26.9%	(24.2–29.7)	34.4%	(30.1–39.0)
- CDS without end-of-life decision	1.6%	(1.2–2.2)	3.2%	(2.2–4.5)	5.1%	(3.4–7.6)
- CDS combined with end-of-life decision	22.8%	(21.1–25.6)	23.7%	(21.2–26.5)	29.3%	(25.2–33.8)

^a^100% = all non-sudden expected deaths; percentages weighted to region-sex-age-specific response rates

### Place of death

As outlined in Table 4, non-sudden expected deaths were more likely to occur without MELDs at home (25.8%) than in hospitals (15.7%) or nursing homes (17.6%) in the German-speaking region (Table 4). Forgoing life-prolonging treatment was less frequent at home than in hospitals or long-term care homes in the German-speaking region, and less frequent at home than in hospitals in the French-speaking region. Intensified alleviation of symptoms showed similar prevalence in all places of death in the French- and Italian-speaking regions, but was more frequent in hospitals (68.8%) and less frequent for home (53.1%) deaths than in long-term care homes (62.7%) in the German-speaking region. Assisted suicide was more frequent at home in the German- (4.2%) and French-speaking (9%) regions, and did not occur in our sample in the Italian-speaking region.

**Table 4:** Prevalence of medical end-of-life practices, Switzerland 2013, by language region and place of death

Regions	German-speaking		French-speaking		Italian-speaking	
Number of non-sudden expected deaths (eligible for end-of-life decision)	*N* = 2256		*N* = 992		*N* = 430	
	%^a^	95% CI	%^a^	95% CI	%^a^	95% CI
At home	*N* = 265		*N* = 96		*N* = 67	
	*11.6%*	*(10.4–13)*	*9.5%*	*(7.9–11.5)*	*15.6%*	*(12.4–19.3)*
No end-of-life practice	25.8	(20.9–31.4)	24.6	(17.0–34.2)	31.1	(21.1–43.2)
Forgoing treatment total	60.3	(54.2–66.0)	47.0	(37.2–57.1)	45.8	(34.2–57.8)
Alleviation of pain & symptoms total	53.1	(47.1–59.1)	60.2	(50.0–69.6)	63.1	(51.0–73.8)
vPhysician-assisted death total	5.7	(3.5–9.2)	15.4	(9.3–24.4)	1.6	(0.2–10.5)
- Assisted suicide	4.2	(2.3–7.5)	9.0	(4.6–17.0)	–	
In long-term care homes	*N* = 982		*N* = 371		*N* = 186	
	*44.3%*	*(42.3–46.4)*	*38.6%*	*(35.6–41.7)*	*43.1%*	*(38.5–47.8)*
No end-of-life practice	17.6	(15.3–20.1)	23.9	(19.8–28.5)	27.1	(21.2–34.0)
Forgoing treatment total	71.5	(68.6–74.3)	59.1	(53.9–64.0)	54.8	(47.6–61.8)
Alleviation of pain and symptoms total	62.7	(59.6–65.7)	63.2	(58.2–68.0)	63.1	(55.9–69.8)
Physician-assisted death total	1.2	(0.7–2.2)	3.0	(1.7–5.4)	3.1	(1.4–6.8)
- Assisted suicide	0.1	(0.0–0.8)	0.3	(0.0–2.0)	–	
In hospital	*N* = 973		*N* = 522		*N* = 174	
	*42.5%*	*(40.5–44.6)*	*51.5%*	*(48.4–54.7)*	*40.6%*	*(36.1–45.4)*
No end-of-life practice	15.7	(13.5–18.1)	26.0	(22.4–30.0)	22.7	(17.0–29.5)
Forgoing treatment total	73.6	(70.4–75.9)	62.6	(58.4–66.7)	64.5	(57.1–71.3)
Alleviation of pain and symptoms total	68.8	(65.8–71.6)	60.2	(55.9–64.4)	65.9	(58.6–72.6)
Physician-assisted death total	2.0	(1.3–3.1)	1.7	(0.9–3.2)	0.5	(0.1–3.8)
- Assisted suicide	–		0.4	(0.1–1.4)	–	

^a^100% = all non-sudden expected deaths; percentages weighted to region-sex-age-specific response rates

Data in this table include cases in which more than one end-of-life decision were taken

### Shared decision-making

Most MELDs were discussed with the patient or relatives, or based on previously known patient wishes (Table 5), with only a minority being discussed with the patient at the time of the decision. Patient involvement was less frequent in the Italian-speaking region (16%), as compared to the French- (31.2%) and German-speaking (35.6%) regions. Even when patients were fully capable of decision-making, up to 40% of MELDs occurred without their involvement, and approximately 12% occurred even without involving their relatives and without knowledge of previously expressed patient wishes.

**Table 5:** Discussion of medical end-of-life decisions^a^ in function of the patient’s decision-making capacity, Switzerland 2013, by language region

Regions	German-speaking		French-speaking		Italian-speaking	
Deaths with end-of-life practice mentioned (eligible for involvement)	*N* = 1856		*N* = 744		*N* = 318	
	%^b^	95% CI	%^b^	95% CI	%^b^	95% CI
Discussed with patient	35.6%	(33.4–37.5)	31.2%	(27.9–34.6)	16.0%	(12.4–20.4)
Patient fully capable	73.3%	(69.6–76.6)	71.2%	(65.3–76.5)	60.0%	(47.2–71.6)
Patient not fully capable	37.1%	(32.3–42.3)	30.3%	(22.9–38.8)	8.4%	(3.8–17.6)
Patient not capable at all	9.7%	(7.6–12.2)	5.8%	(3.4–9.6)	6.9%	(3.6–12.8)
Patient’s capacity unknown	3.4%	(1.7–6.7)	–		–	
Discussed with patient and/or relatives and/or patient ever expressed wish	76.5%	(74.5–78.4)	73.8%	(70.5–76.9)	69.0%	(63.7–73.8)
Patient fully capable	87.8%	(85.0–90.2)	88.5%	(84.0–91.9)	87.4%	(76.5–93.6)
Patient not fully capable	85.3%	(81.2–88.6)	82.9%	(75.3–88.5)	75.6%	(64.4–84.1)
Patient not capable at all	79.8%	(76.5–82.7)	82.2%	(76.6–86.6)	82.8%	(75.3–88.4)
Patient’s capacity unknown	22.1%	(17.2–28.0)	20.4%	(14.2–28.4)	13.1%	(6.6–24.2)

^a^CDS is not a MELD and is thus not included in this table

^b^100% = all deaths with reported end-of-life practice; percentages weighted to region-sex-age-specific response rates

## Discussion

To our best knowledge, this is the first population-level death-certificate study allowing comparison of real practice MELDs other than euthanasia between different cultural regions within the same country. Our study shows that MELDs are more frequent overall in the German-speaking region, and the prevalence of the MELD deemed most explicit varies between the three language regions in Switzerland, supporting the view that cultural differences subsist under the same legal system [2]. The view of Switzerland as “Europe in miniature” [16] is at least partly corroborated by our results. International comparison nevertheless shows a generally high proportion of MELDs in all regions of Switzerland when compared to countries sharing a language, such as France [19] or Italy [11], with no data currently available on the practice of MELD in Germany or Austria. This is in line with other studies suggesting that Switzerland is among the European countries where patient autonomy is given a greater weight in MELDs [20, 21].

Previous studies have shown distinct national cultures of end-of-life care [22]. Cultural influence on physicians’ views of MELDs within the same country have been reported in a former Swiss survey [16] as well as between Walloon and Flemish physicians in Belgium [2]. Differences in patients’ and families’ requests may have even more impact than physicians’ attitudes [20]. Despite being limited to a single specialty, reports of practices by a Sentinel Network of General Practitioners in the Dutch- and French-speaking regions of Belgium had also shown that the prevalence of MELDs was higher in the Dutch- than in the French-speaking community [23].

Our data suggest greater reluctance in forgoing life-prolonging treatment in the French- and Italian-speaking regions than in the German-speaking region, with partial replacement through either intensified alleviation of symptoms or CDS in French- and Italian-speaking regions. This is consonant with data from Belgium, showing more negative attitudes towards euthanasia and lower rates of reporting from the French- as compared to the Dutch-speaking region [2], and higher prevalence of CDS among French- than among Dutch-speaking physicians in the Brussels area [24]. In contrast, variation of intensified alleviation of symptoms between regions was almost absent in our sample. It has been proposed that this could be due to a perception that this constitutes a more direct response to a clinical situation [20]. There were no significant differences in the practice of suicide assistance in the German- and French-speaking regions, yet our sample recorded no case of suicide assistance in the Italian-speaking region [25].

International comparisons show a generally high proportion of MELDs in all regions of Switzerland. Although these data were collected at different times, overall prevalence for MELDs was somewhat lower in France than in the French-speaking region [19] and substantially lower in Italy than in the Italian-speaking region of Switzerland [11]. Further, forgoing life-prolonging treatment was somewhat less frequent, and intensified alleviation of symptoms somewhat more frequent, in France than in the French-speaking region of Switzerland [19]. No comparative data are available from Germany.

Greater reluctance to forgo life-prolonging treatment may not be due to identical factors in the French- and Italian-speaking regions. The observed greater prevalence of hospital deaths in the French-speaking region suggests a greater tendency to pursue treatment, a finding consonant with greater appreciation of curative, technological, and specialist medicine in the French-speaking than in the Dutch-speaking community in Belgium [23]. Additionally, it is also consistent with a health system focus on hospitals rather than nursing homes in the French-speaking region, and with data showing structural effects of health systems on place of death [26]. However, this cannot explain the greater reluctance in the Italian-speaking region, where more deaths occurred at home than in the French-speaking region.

That most assisted suicide found in our sample took place at home is consistent with many hospitals’ reluctance to allow suicide assistance, but also with data suggesting that the key reasons for patients to choose their homes include a concern to avoid loss of control [25].

Patient involvement in decisions was less frequent in the Italian-speaking region. Although this was mostly the case for patients deemed “not fully capable”, a similar trend was shown for fully capable patients and those allocated to this category represented an implausibly small proportion when compared to the other two language regions. These data suggest that Italian-speaking doctors may be loath to discuss MELD, possibly because they have remained ambivalent towards MELDs, as suggested by previous findings comparing attitudes [16]. This is also consistent with international data showing higher prevalence of treatment preference discussions with Belgian and Dutch than with Spanish and Italian patients [27]. We did not find differences in the involvement of patients in the German- and French-speaking regions. However, a comparison of patient involvement in the Flemish- and French-speaking regions of Belgium did not show significant differences in patient involvement either [23].

Although culture is being used as an “umbrella term” herein, encompassing many different elements, our findings are compatible with the view that, compared to the German-speaking region, a more family-oriented approach is prevalent in the Italian-speaking region and a more technology-oriented approach in the French-speaking region. End-of-life decisions are debated simultaneously within countries, within single-language transnational regions, and internationally. Although these differences require qualitative exploration in order to be better understood, our results may show how national and language-based discussions can interact.

Our study has several limitations. The optimal phrasing of the questionnaire for international comparison purposes remains controversial [28]; this, however, is unlikely to affect intra-national comparison. We cannot exclude the possibility of a non-response bias, especially since responding that death was sudden and unexpected offered an easy option to skip all potentially sensitive questions. This would not have affected the intra-national comparison, were it not for the fact that response rates were different in the three language regions. This effect is not likely to be large; indeed, response rates in our study were remarkably high, especially given the fact that our survey had no official monitoring mission. Even in the French-speaking region, where response rates were lowest, they were clearly higher than in France (40%) [19] and Italy (44%) [11]. We may nevertheless have underestimated the prevalence of MELDs. The observation unit was deaths and not physicians; several physicians filled in more than one questionnaire. Due to the anonymous nature of the survey, questionnaires stemming from the same physician could not be identified. Therefore, the results are not necessarily representative for Swiss physicians. Although our sample size was much larger than other similar studies [23], small differences in the prevalence of MELDs may nevertheless have escaped our sample size, especially in the smaller language regions. More importantly, this method only allows exploration of what physicians believe happened. Depending on respondents’ technical knowledge regarding MELDs, their beliefs may sometimes have been mistaken [29]. Despite this, this kind of study is still widely accepted as the gold standard for assessing MELDs on a population level.

## Conclusion

Differences within Switzerland partly reflect practices in countries with the same linguistic tradition, but international comparisons show a generally high proportion of MELDs in all areas of the country, in line with the greater role given to patient autonomy. Our findings show how cultural contexts and legislation can interact in shaping the prevalence of MELDs.
